# High resolution dynamic ultrasound atlas of embryonic and fetal development of the common marmoset

**DOI:** 10.1007/s10815-024-03072-2

**Published:** 2024-03-06

**Authors:** Rohan R. Soman, Margaret M. Fabiszak, Michael McPhee, Peter Schade, Winrich Freiwald, Ali H. Brivanlou

**Affiliations:** 1 Tri-Institutional MD-PhD Program, Weill Cornell Medical College, New York, NY USA; 2 Laboratory of Synthetic Embryology, Rockefeller University, New York, NY USA; 3 Laboratory of Neural Systems, Rockefeller University, New York, NY USA

**Keywords:** Ultrasound, Marmoset, Atlas, Development, Embryology

## Abstract

**Purpose:**

The common marmoset (*Callithrix jacchus*) provides an ideal model to study early development of primates, and an *in viv*o platform to validate conclusions from in vitro studies of human embryos and embryo models. Currently, however, no established staging atlas of marmoset embryonic development exists. Using high-resolution, longitudinal ultrasound scans on live pregnant marmosets, we present the first dynamic in vivo imaging of entire primate gestation beginning with attachment until the last day before birth.

**Methods:**

Our study unveils the first dynamic images of an in vivo attached mammalian embryo developing in utero, and the intricacies of the delayed development period unique to the common marmoset amongst primates, revealing a window for somatic interventions.

**Results:**

Established obstetric and embryologic measurements for each scan were used comparatively with the standardized Carnegie staging of human development to highlight similarities and differences. Our study also allows for tracking the development of major organs. We focus on the ontogeny of the primate heart and brain. Finally, input ultrasound images were used to train deep neural networks to accurately determine the gestational age. All our ultrasounds and staging data recording are posted online so that the atlas can be used as a community resource toward monitoring and managing marmoset breeding colonies.

**Conclusion:**

The temporal and spatial resolution of ultrasound achieved in this study demonstrates the promise of noninvasive imaging in the marmoset for the in vivo study of primate-specific aspects of embryonic and fetal development.

**Supplementary Information:**

The online version contains supplementary material available at 10.1007/s10815-024-03072-2.

## Introduction

The common marmoset (*Callithrix jacchus*) provides an ideal model for primate studies. Given their small size of ~ 350 g, the ability to easily establish large breeding colonies in captivity while preserving naturalistic social structures, and the propensity to produce 2 L per year, the marmoset is an ideal system to study early primate development, in vivo, a prospect that is ethically impossible to contemplate in humans, even if it were to be technically feasible [1–4]. Recent advances at the tissue and cellular level, including self-organizing human embryonic stem cells (hESCs) into embryoids and organoids, combined with the advanced molecular tools of genome editing and transgenic technologies are paving the way forward the exploration of primate-specific aspects of embryonic development. Yet these in vitro results will ultimately have to be tested in vivo to validate the relevance of these findings. With a gestation period of 140–150 days most often producing dizygotic twins, the common marmoset possesses numerous benefits for developmental studies and creating transgenic primates [5–9]. There is an essential need to establish an accessible and scalable means by which to monitor embryonic and fetal development with demonstrated correlates to ex vivo references.

Ultrasound approaches and ex vivo histological studies have been previously used to follow organ development and fetal growth curves and their correlation with the classical Carnegie stages [10–14]. However, they lacked the temporal resolution to capture all stages of development and the spatial resolution to adequately visualize relevant embryonic structures. Reversely, while ultrasound approaches have been used in the past to characterize in vivo embryonic development in humans [15–18], this has not been explored in the marmoset. A major limiting factor in live embryo imaging in humans is that pregnancy cannot be detected until 3–4 weeks of gestation and thus ultrasound most commonly occurs after week 5 [19]. Additionally, patients rarely undergo full ultrasound exams more often than once per trimester, making longitudinal studies impossible. In addition to the fundamental knowledge of embryogenesis, insights into primate-specific embryonic development are of critical clinical importance as the incidence of first trimester, early pregnancy loss in humans approaches 30% [20]. Nearly 50% of these losses are thought to stem from chromosomal abnormalities, many with unknown functional consequences, and the remaining etiology remains ambiguous and difficult to study in animal models which lack uniquely primate uterine, embryonic, and extraembryonic structures [21].

During later stages of development, organogenesis has been evaluated grossly by ex vivo studies that measured the emergence and growth of organs [3]. Magnetic resonance imaging (MRI) has also been used in the past to study the development of specific organs such as the brain, both in vivo and ex vivo [22, 23]. MRI, however, requires a significant investment in custom and costly equipment thereby complicating tracking individual fetuses to detect key developmental milestones. Furthermore, although the spatial resolution in these MRI studies proved to be exquisite, temporal resolution and movement artifact limits the measure of dynamic events. These include, for example, heartbeat or blood flow that are milestones of cardiac development. Here we present a high-resolution, serial ultrasound atlas for embryonic and fetal marmoset development that establishes in vivo correlates to ex vivo staging, reveals insights into the period of delayed development as well as key features of organogenesis, and provides the basis for automating developmental staging with machine learning techniques.

## Results and discussion

### In vivo detection of the earliest stages of implantation in a primate embryo

**Fig. 1:**
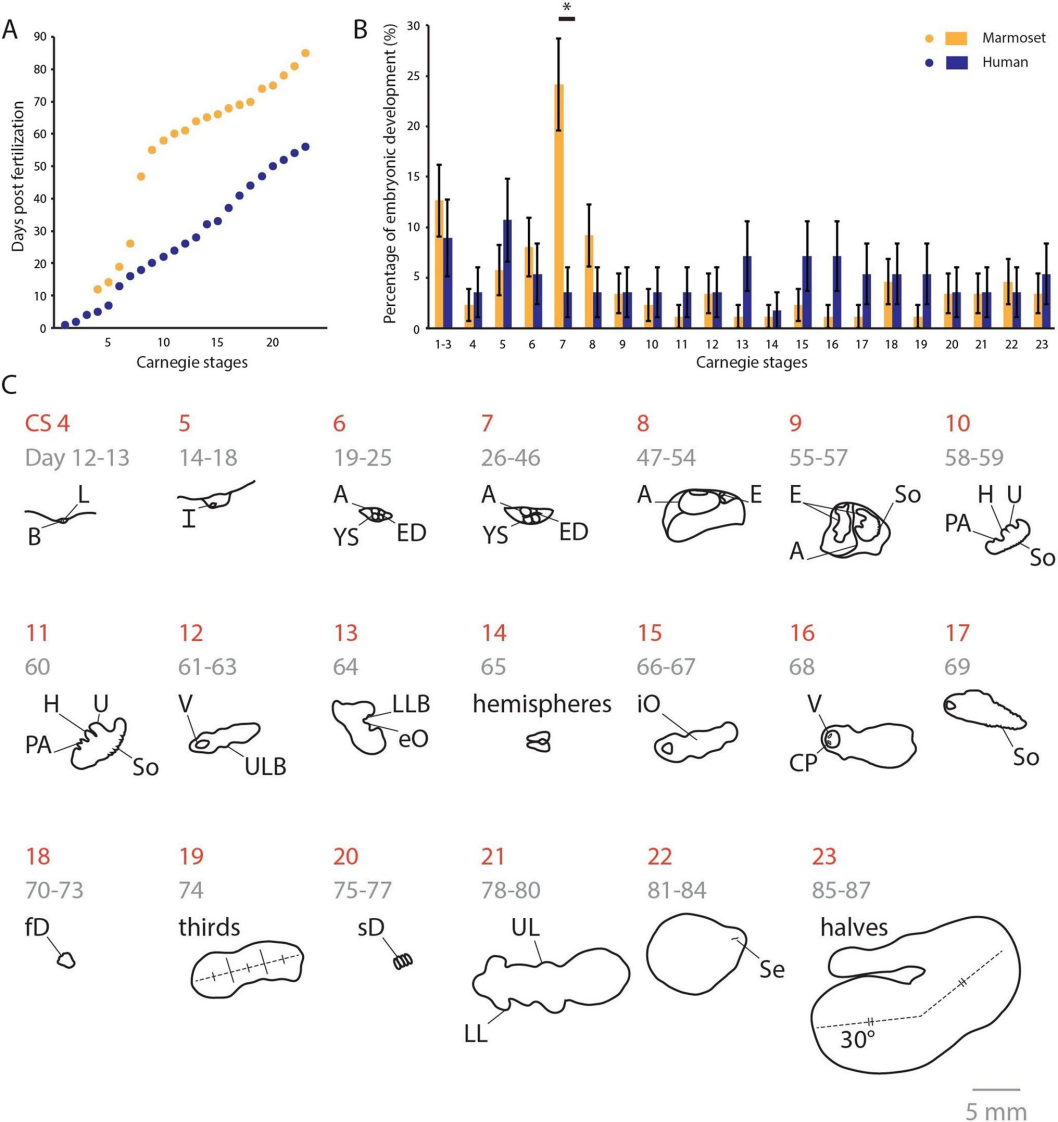
Early ultrasound measurements reveal Carnegie Stages. **A** The first day postfertilization during which the developing embryo enters into each Carnegie Stage (orange, marmoset; blue, human). Human data is taken from O’Rahilly and Muller, 2010 [27]. **B** The percentage of the embryonic development (estimated to be 87 days marmoset, 56 days human) spent in each Carnegie Stage. **C** Representative ultrasound images taken for each Carnegie Stage. Outlines in black reveal staging features relevant in ultrasounds. Dotted lines highlight key areas or orientations in figures. Red numbers indicate Carnegie Stage. Gray numbers indicate the range of days per stage. A, amnion; B, blastocyst; CP, choroid plexus; E, embryo; eO, external organs; ED, embryonic disk; fD, fused digits; H, heart; I, implantation; iO, internal organs; L, lumen; LL, lower limb; LLB, lower limb bud; PA, pharyngeal arches; sD, separated digits; Se, septum; So, somites; U, umbilicus; UL, upper limb; ULB, upper limb bud; V, ventricle; YS, yolk sac

IVF approaches have allowed the characterization of marmoset preimplantation embryos at the cellular and molecular level [1, 24]. These in vitro studies revealed that development from fertilization to blastocyst stage is slower than in humans (9–13 days in marmosets versus 5–7 days in humans). While the cause for this delay remains unknown, it does not represent diapause as it is not dependent on lactation [25], but rather slow growth due to metabolic demand for the twinned blastocysts to develop a fused chorion [26]. Information on post-implantation embryos, on the other hand, remains sparse and is mostly based on ex vivo histological studies and low-resolution ultrasound acquired on long-time intervals. In order to generate a high-resolution atlas of developmental stages of *Callithrix jacchus*, we tracked 7 dams through 13 pregnancies for a total of 34 fetuses. After confirming pregnancy, each fetus was tracked twice per week for the first half of the pregnancy and weekly thereafter for an average of 19 ultrasound sessions per fetus, using longitudinal, high-resolution imaging. Pooling scans across all of our subjects, a daily coverage of 93% of embryonic and 82% of fetal development was used to classify embryos using the Carnegie Staging method. Our earliest images captured at day 12 postfertilization unveiled the morphology of a newly attached embryos in vivo: a first in mammalian and primate embryology (Fig. 1).

### Protracted gastrulation in marmoset

In order to compare the timing of embryogenesis to human Carnegie Stages, we plotted the amount of time it takes for a marmoset embryo to reach the equivalent stage to that of humans, using human data obtained from O’Rahilly and Muller, 2010 [27] at a time resolution previously impossible (Fig. 1A). Given that marmoset gestation is approximately half that of human gestation, we would have expected marmosets to enter each stage of embryonic development earlier than humans. Surprisingly, however, plotting the days postfertilization in function of Carnegie stages, we find that protracted development occurred during one distinct stage (Carnegie Stage VII) at day 20 to 50 postfertilization (Fig. 1A). This was also highlighted by our analysis of the amount of time embryos spent in each stage and confirmed that *only* within this stage does the marmoset embryo spend significantly more time than human embryos as measured by a two-proportion *z*-test (*p* = 0.0038) (Fig. 1B). We summarize representative features used to classify each of the 23 Carnegie Stages in Fig. 1C that are based on a total of 289 individual ultrasounds recorded across marmoset embryonic development presented in Supplemental Figs. 1 and 2.

**Fig. 2:**
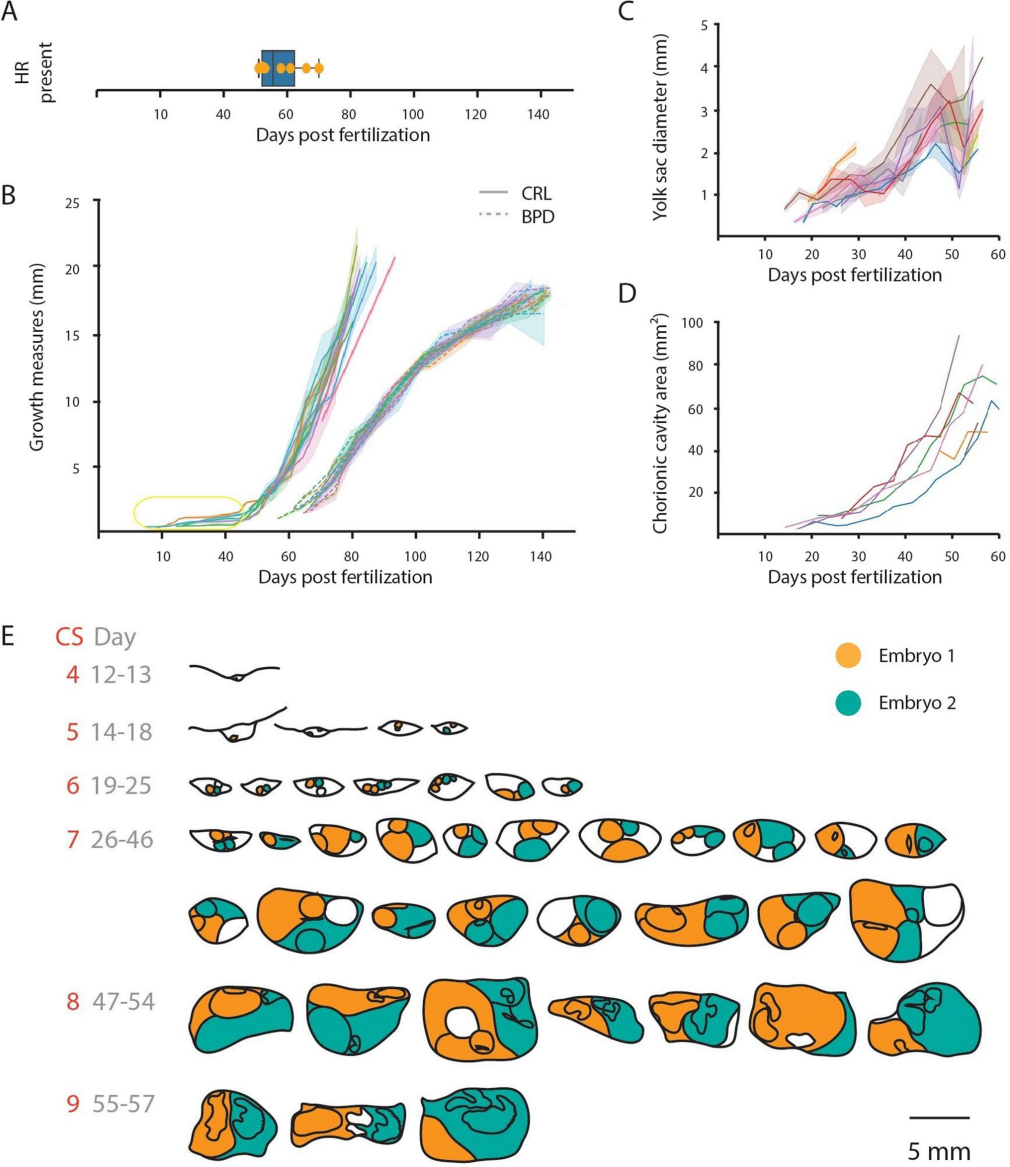
High-resolution, serial ultrasounds reveal period of protracted embryonic development distinct from diapause during gastrulation. **A** First measurable heart rates in embryos across seven pregnancies (mean = day 57, 95% CI = day 52–64). **B** Classical clinical embryonic measurements for crown-rump length (CRL) from day 14 to 93 and biparietal distance (BPD) from 87 days to birth. Measurements are taken across all embryos during 7 pregnancies. Each colored line corresponds to one course of pregnancy; the width of shading represents the 95% confidence interval for each embryo in the pregnancy. Inset shows range of values for C, D. **C** Yolk sac diameter measured from day 13–53 days before birth. **D** Maximum chorionic cavity area was calculated from 13 to 53 days before birth as a correlate of chorionic volume. **E** Relevant outlines of serial embryonic ultrasounds across all pregnancies to maximize coverage. Red numbers indicate Carnegie Stage. Gray numbers indicate range of approximate days postfertilization. Colored shading indicates twins in each image. Remaining white space is extra-amniotic or a triplet

### Dramatic remodeling of extraembryonic tissue during the protracted period

To further characterize marmoset early embryonic growth and development, we tracked the time of the first discernable heartbeat occurring at day 57 (Fig. 2A), measured the size of the embryonic disk as reflected by the crown-rump length (CRL) from day 14 to 93, and biparietal distance (BPD) from day 87 to birth across 35 fetuses (Fig. 2B). This revealed a period in which the CRL changes little over nearly a 40-day period, after which a rapid period of growth begins that temporally coincides with the first heartbeat. Interestingly, however, examination of individual ultrasound scans revealed that the embryos and the uterus are not in a quiescent state during this 40-day latency. In fact, dramatic extraembryonic growth and restructuring is taking place during this time. Measurements of maximum chorionic cavity cross-sectional area and yolk sac diameter taken during days 14–62 confirmed the dramatic morphologic changes occurring in extraembryonic tissues during this time (Fig. 2C and D). The relative changes in tissue distribution of the lumen, embryo, yolk sac, and amnion during each relevant stage are depicted in Fig. 2E and are based on original ultrasounds shown in Supplementary Fig. 1.

This fact distinguishes the developmental delay observed in the marmoset from that of the other periods of mammalian developmental delay such as diapause, which is strictly defined as a quiescent period with no growth during the blastocyst stage prior to implantation. Marmosets do not delay pregnancies based on season, resource availability, or hormonal state [26], but nevertheless, the embryos exhibit marked delay in progressing through the stages of embryonic development. Thus, we propose that this pause in embryogenesis reflects a normal stage of marmoset development that has not been shown to exist so far in any other primate. Additionally, this delayed development of the embryonic compared to extraembryonic tissue reveals a window of opportunity to interrogate and manipulate the mechanisms of early primate development in vivo by using emerging molecular techniques.

### Standard gestational measures as predictors of fertilization age

We next sought to determine which gestational measures, if any, are reliable predictors of the day of fertilization. Data from each measure were fit to a polynomial linear regression (Supplemental Fig. 3). All measures proved useful predictors with 95% confidence intervals of approximately 10 days, with chorionic cavity area as the best predictor of early embryonic age (days 13–53) and BPD as the best overall predictor (days 87-birth) (Fig. 2B). Previous studies suggest that BPD measurements diverge as an indicator of postnatal survival [11]. We see no such separation within our BPD measures. In fact, due to the lower variance of measures of BPD [10], we find them to be a better predictor of gestational age and due date.

**Fig. 3:**
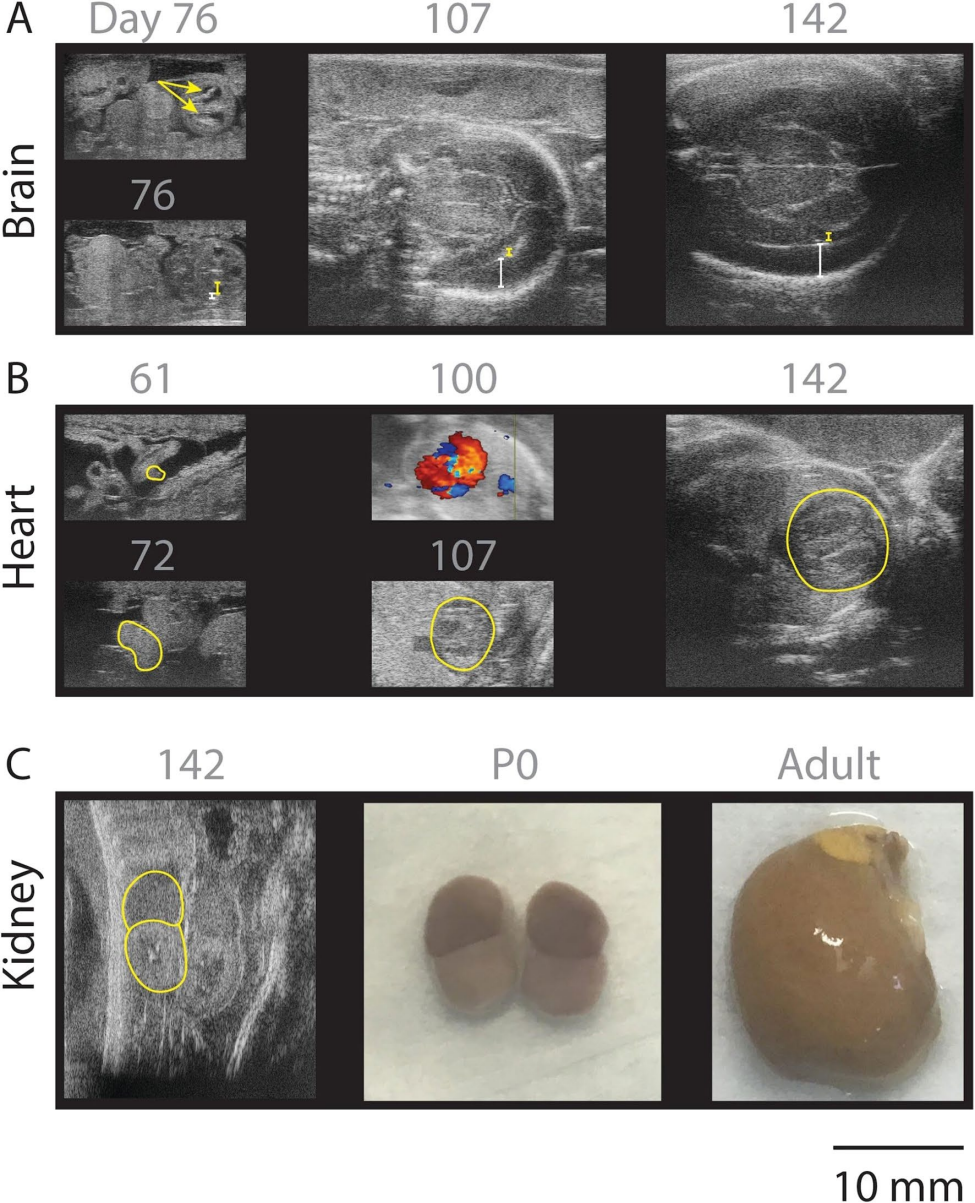
Organogenesis milestones tracked with ultrasound. **A** Brain development. Choroid plexus distinct within lateral ventricles as denoted by arrows. Large lateral ventricles (yellow bars) that gradually reduce in size as the cortical thickness increases over time (white bars). Days 76–107 are coronal views of the brain. Day 142 is a horizontal view. **B** Development of the heart tube with subsequent inclusion into the thoracic cavity and development of distinct chambers. Yellow outlines indicate regions of heart development. Day 100 is a Doppler scan of the heart in which blue and red indicate blood moving in different directions. **C** (i) Adrenal to kidney ratio on ultrasound reveals ratio of 1:1 shown to be accurate with (ii) ex vivo gross dissection. (iii) This ratio dramatically decreases as the kidneys and adrenals mature. All gray text indicates days postfertilization except for P0 which is day of birth and adult

### Fetal organogenesis milestones: the brain

Throughout this ultrasound atlas, we delineate key features of organogenesis specifically focusing on the brain, heart, and kidneys. Our ultrasound analysis can detect the onset of primate neurulation in vivo which begins with the emergence of the most dorso-anterior telencephalic structures at neural plate stages (Carnegie Stage 8, 47 days after fertilization), concomitantly with morphogenetic events that fold the neural plate into a neural tube (Carnegie Stage 10, 58 days after fertilization, Supplementary Fig. 2). Tracking brain development in vivo, we noticed unusually enlarged lateral ventricles measured to be 0.82 mm at the most ventral portion of the lateral ventricles found in a coronal section and a thin cortical mantle 0.38 mm (Carnegie Stage 20, 76 days postfertilization, Fig. 3A). These large ventricles gradually shrink in size, ultimately reaching a diameter of 0.41 mm only 31 days later at 107 days postfertilization. The cortical mantle, on the other hand, expands to reach a diameter of 2.14 mm at 107 days postfertilization, as the ventricular zone gives rise to the progenitor cells which differentiate into the layered cortex (Fig. 3A, middle panel). These findings are in agreement with early histological studies [22] and follow the same trend of developmental timing as in human development [28]. The fact that primate cortical layer development can be observed in vivo in real time, with a relatively simple and widely accessible ultrasound technique holds the promise of connecting the origin of the brain to its function during adulthood.

### Fetal organogenesis milestones: the heart

One of the first signs of a viable fetus is the presence of a heartbeat. The heart which is derived from the dorsal mesoderm during gastrulation begins as a tube of 0.81 mm in length and 0.42 mm in width at Carnegie Stage 12, 61 days postfertilization (Fig. 3B, left, top). The tube then undergoes a spectacular folding process, ultimately generating the 4 chambers of a functional heart by Carnegie Stage 18, 72 days postfertilization (Fig. 3B, left, bottom). We first detected heartbeat in the marmoset fetus at day 57 (± 3.8), which corresponds to estimates of first heartbeats as measured in days postfertilization occurring in Carnegie Stage 9 or 10 in human studies [29].

### Fetal organogenesis milestones: adrenal and kidney development

Tracking the development of the kidney yielded another surprise. The first morphological signs of the emergence of the pronephros (the anlage of kidney and adrenal glands) should occur at Carnegie Stage 14 for the kidney and 18 for the adrenal [30] as per human studies. Throughout the remainder of development, it is contested whether or not the two organs maintain a ratio of adrenal:kidney size of roughly 1:4 [31] or if the adrenal surges in size achieving a ratio of 1:1 [32]. We demonstrated here that the marmoset ratio of adrenal:kidney size reaches 1:1 just before birth as measured by ultrasound (Fig. 3C, left), is confirmed by ex vivo gross dissection shortly after birth (Fig. 3C, middle), and then experiences a transition of adrenal shrinkage and kidney growth reaching a ratio of roughly 1:8 by adulthood (Fig. 3C, right). This finding is in line with the second of the two human hypotheses and thereby forms a model in which this process of growth and shrinkage can be molecularly interrogated.

### A deep network model of marmoset development to predict marmoset embryonic age

While ultrasound has proven to be an invaluable method of investigating the anatomy and physiology of embryonic and fetal development, there remain several caveats. Firstly, proper scanning and interpretation of ultrasound imaging require a skilled imaging technician and analyst, in both non-human primate and in clinical human radiology. Secondly, accurate and precise measures of embryonic age and stage of development, especially early in development, require multiple serial scans — which is inconvenient in the experimental setting and infeasible in the clinic. We used the data collected as part of this ultrasound atlas to generate predictive models of marmoset development using machine learning methods, allowing accurate extrapolation of embryonic age from single random frames. In doing so, we provide a tool that minimizes the technical expertise, time, and effort required to track marmoset development.

**Fig. 4:**
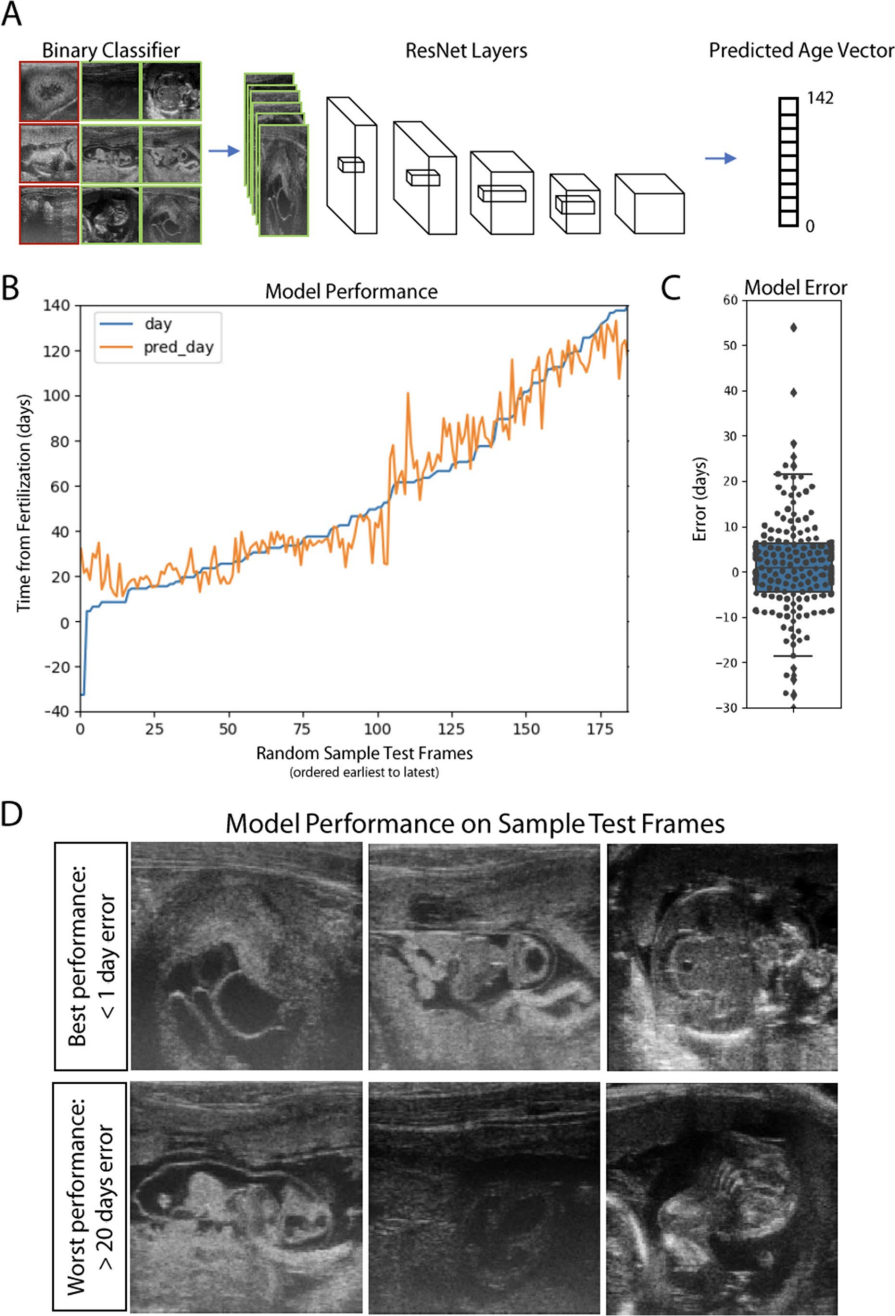
A deep learning network model of marmoset development predicts embryonic age. **A** Schematic of the deep network model. Images are classified by a binary classifier trained on outputs from ResNet50. Images labeled as salient are input for a separate deep network trained to predict time until birth. **B** Performance of model using 180 randomly selected frames as input with predicted age (orange) and actual age (blue) plotted according to estimated time postfertilization. **C** Predictive accuracy of the model as a box-and-whisker plot based upon predicted age for the frames plotted in **B**. Median error = 5.44 days, 95% CI =  ± 8.84 days. **D** Sample frames for which the model performed best (top row) and worst (bottom). Clear anatomic landmarks can be seen in the top frames, while the bottom frames are of poor quality

We utilized the Keras deep learning API [33] to define and train a set of residual neural networks that sort salient frames and assign an embryonic age in an automated fashion (Fig. 4A). When evaluating the performance of this system on a random set of novel, unbiased ultrasound frames (i.e., drawn from any of the frames collected during any of the ultrasound sessions in our database), our models were able to estimate embryonic age with a median error of 5.44 days (95% CI ± 8.84 days, Fig. 4B, C, D). In comparison, clinical measurements of gestational age by a skilled analyst as described in Fig. 2 achieved a maximum accuracy of between ± 8 and 13 days, depending on the measure and timepoint analyzed. This tool also opens new windows for analysis, not only by predicting windows for intervention using a single scan but also including the potential for predicting abnormal development and evaluating health in transgenic animals.‬ ‬‬‬‬‬‬‬‬‬‬‬‬‬

Our high-resolution, serial marmoset ultrasound atlas builds on the work done in this field from the earliest ex vivo studies of embryos [26] through to the first ultrasound-derived growth curves [10, 34] and in utero MRI atlas [22]. This approach provides an opportunity to study marmoset development at the cellular and embryological level and to develop an in vivo platform for genome editing to understand basic development as well as disease modeling with high impacts on our understanding of reproduction, cognition, and biomedical interventions. We have made our ultrasound atlas and resources accessible to the community (CallithrixJaccusAtlas.squarespace.com, password: commonmarmoset). We hope that this site will also serve as a repository for users within the marmoset community for submission of additional ultrasound images for immediate estimations of embryonic age, as well as a crowdsourcing platform to add to the machine learning database. We aim to broaden the range of machines and users to provide a dynamic, comprehensive, and detailed atlas that will continue to be consistently refined and strengthened by the community.

In the near future, genome-edited marmoset embryonic stem cells will be used to generate self-organizing embryo models such as *gastruloids* and *neuruloids* that will be used to dissect early primate embryonic events. This is already used to model early human development. In that context the generation of an isogenic in vivo model that harbors the exact same genetic signature as the gastruloid and neuruloids will provide the unique opportunity to validate in vitro data in the context of a living marmoset. Additionally, genome-edited marmosets will also provide an in vivo platform to validate discoveries made in the human gastruloids and neuruloids, a prospect already on the horizon.

## Methods

### Animals

All animals were in normal health and were group housed with other conspecifics at the facility. All procedures were approved by The Rockefeller University Animal Care and Use Committee and were performed in accordance with the National Institute of Health guidelines for care and use of laboratory animals. The gross dissection of the ex vivo kidneys were conducted on kidneys taken from a pup that did not survive the birthing process and from an adult that was humanely euthanized for the project described in Chapter 2.

### Ultrasound imaging of animals

We performed ultrasound measurements on 7 dams across 13 pregnancies totaling 34 fetuses. Each marmoset was kept in stable, bonded pairs on luetolytic cloprostenol (0.03 mL, 1x/28 days) until entering breeding. Upon commencing breeding, each marmoset was palpated weekly or by ultrasound exam if pregnancy was suspected. Once an open lumen in the uterus was observed, the dam underwent biweekly ultrasound examinations using the Vevo 2100 (FUJIFILM VisualSonics, Ontario, Canada) ultrasound. Probes used included MS700, MS550D, and MS250. Using these three probes, we are able to obtain a range of pixel sizes from 0.01 to 0.04 mm that covered fields of view from 1 to 2 cm wide. For the last half of pregnancy, ultrasounds were conducted on a weekly basis. Ultrasounds were conducted without sedation. Animals were behaviorally trained with positive reinforcement to remain still while being lightly held in a recumbent position.

### Ultrasound analysis

An independent scorer who did not partake in the ultrasound examination took key measurements of crown-rump length and parietal distance for each fetus. Notes were made on stages of organogenesis. Across all animals in the study, there were 104 imaging days consisting of 289 individual ultrasound exams with a total of 6159 recordings. All recordings were exported as DICOM and converted into TIFF stacks using custom MATLAB code (MathWorks, MA, USA). Measurements were made in ImageJ (NIH, MD, USA) and notes were taken in Excel (Microsoft, WA, USA). Data were collected and analyzed in Python (Python Software Foundation, DE, USA) and comparison charts based on notes were made in Excel.

To calculate day postfertilization, we took each animal’s date of birth and assumed a gestation period of 143 days [10]. The day of each ultrasound exam was then converted into a presumed date from fertilization. We plotted all data both in time from birth and presumed day postfertilization. For ease of comparison to other studies, all figures here are plotted only in day postfertilization.

#### Predictive modeling

Image classifier and regression models were built using the Keras deep learning API platform built upon TensorFlow 2.0 in Python [33]. Resnet50 architecture was used to build all models. For binary image classification, a ResNet50 model was fitted with a binary cross-entropy classifier and trained on a set of ~ 200 salient and non-salient frames that were sorted by hand. Following this, we trained deep convolutional dense nets, adapted for usage with ultrasound frames and written in Keras for regression of input frames along the axis of embryonic age, utilizing the time until birth as ground truth. Learning rate and batch size parameters were optimized. We trained on a set of 1200 salient frames with a batch size ranging from 70 to 30 training:validation split, before generalizing the regression model by training across all 800,000 frames from the ultrasound atlas dataset. The Adam optimization scheme was utilized, with learning rates between 0.001 and 0.00001 and batch sizes from 32 to 256, decreasing learning rates by 10^−1^ and doubling batch size after every 25 epochs. We found that performance increased when two separate networks were trained — one for regression during the first half of gestation and another for the second. Validation was performed on a set of 180 randomly selected unseen images.‬‬

##### Declarations

Ali H. Brivanlou: Rumi Scientific, SAB Member.

### Supplementary Information

Below is the link to the electronic supplementary material.

Supplementary file1 (DOCX 1518 KB)

## Data Availability

Data that support our study is openly available in CallithrixJaccusAtlas.squarespace.com, password: commonmarmoset.
