# Supplementary material for: High resolution dynamic ultrasound atlas of embryonic and fetal development of the common marmoset
Source: J Assist Reprod Genet. 2024 Mar 6;41(5):1319–28. doi: 10.1007/s10815-024-03072-2 (PMC11143105; doi:10.1007/s10815-024-03072-2)
Supplement: Supplementary file 1 — Supplementary file1 (DOCX 1518 KB) [file 10815_2024_3072_MOESM1_ESM.docx]

**Supplementary Figure 1. Daily ultrasound images with key features, pre neural tube closure**

Carnegie Stages preceding the closure of the neural tube. Outlines in white reveal the key features for staging. Yellow highlights key areas or orientations in figures. Red numbers indicate Carnegie Stage. Gray numbers indicate the estimated day post fertilization of the image. A. Individual ultrasound images taken from all days sampled for these stages. B. Selected days expanded to show detail.


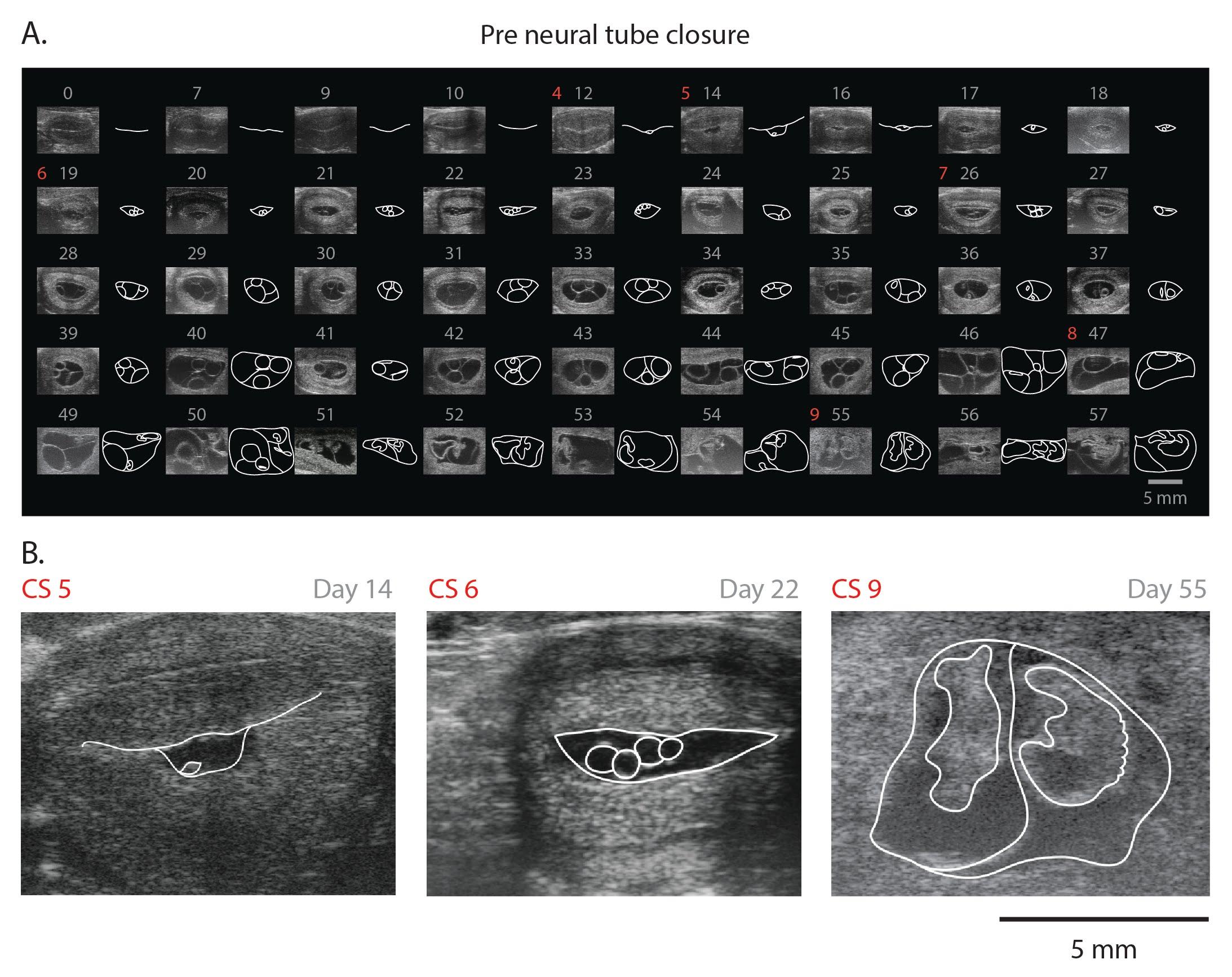


**Supplementary Figure 2. Daily ultrasound images with key features, post neural tube closure**

Carnegie Stages after the closure of the neural tube. Conventions follow Fig 4.2. A. Individual ultrasound images taken from all days sampled for these stages. B. Selected days expanded to show detail. CP, choroid plexus; E, eye; eO, external organs; fD, fused digits; H, heart; iO, internal organs; LL, lower limb; LLB, lower limb bud; PA, pharyngeal arches; sD, separated digits; Se, septum; So, somites; U, umbilicus; UL, upper limb; ULB, upper limb bud; V, ventricle.

**
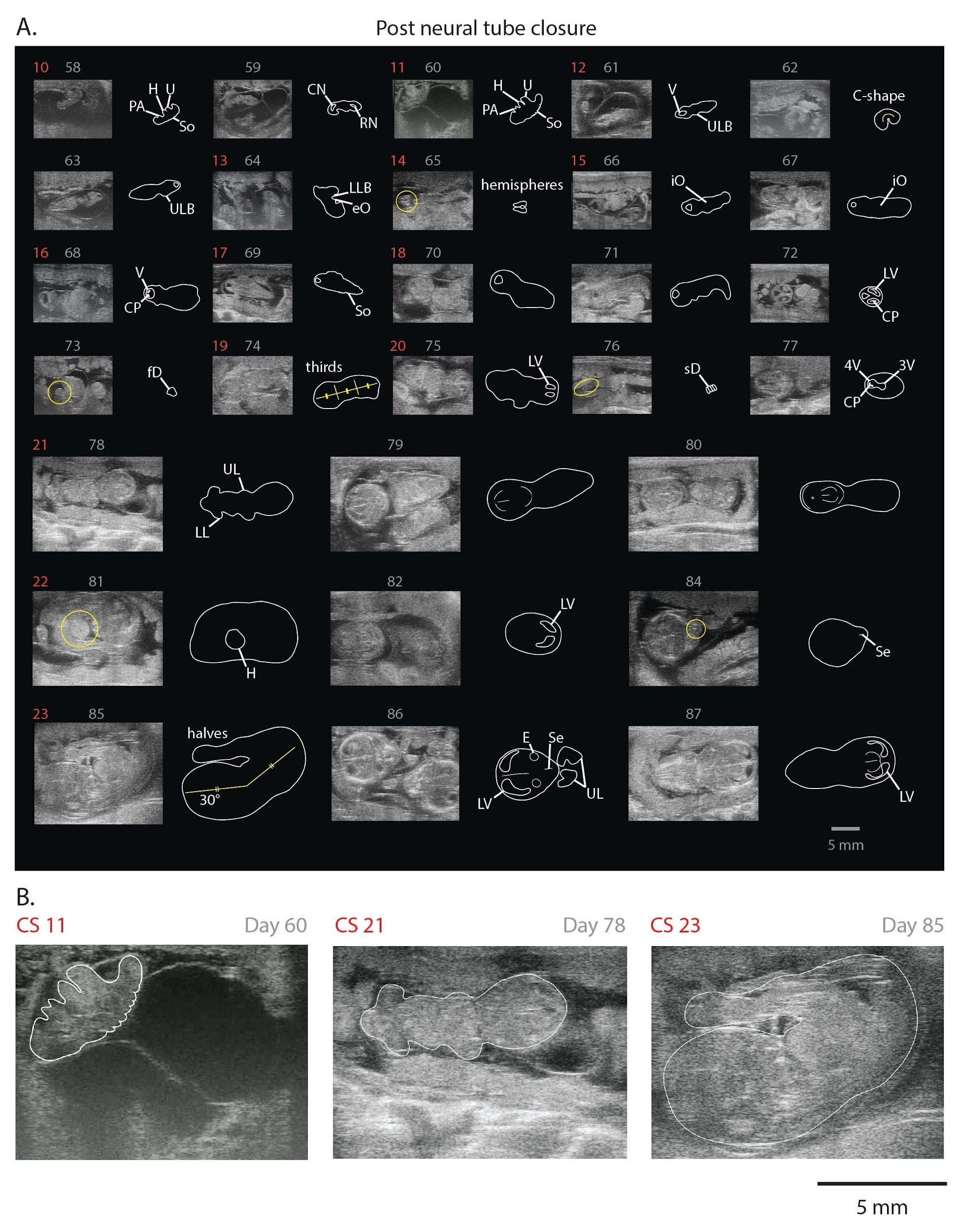
**

**Supplementary Figure 3. Gestational measures as predictors of day of fertilization.**

A. Linear regressions based upon each of chorionic cavity area (CCA), yolk sac diameter (YSD), crown-rump length (CRL), and biparietal diameter (BPD) as individual predictors of fertilization age. Dots represent each measurement aggregated from collected data, and lines trace the curve of best fit. B. Model description and statistics for linear regression model performance for each measure.

**
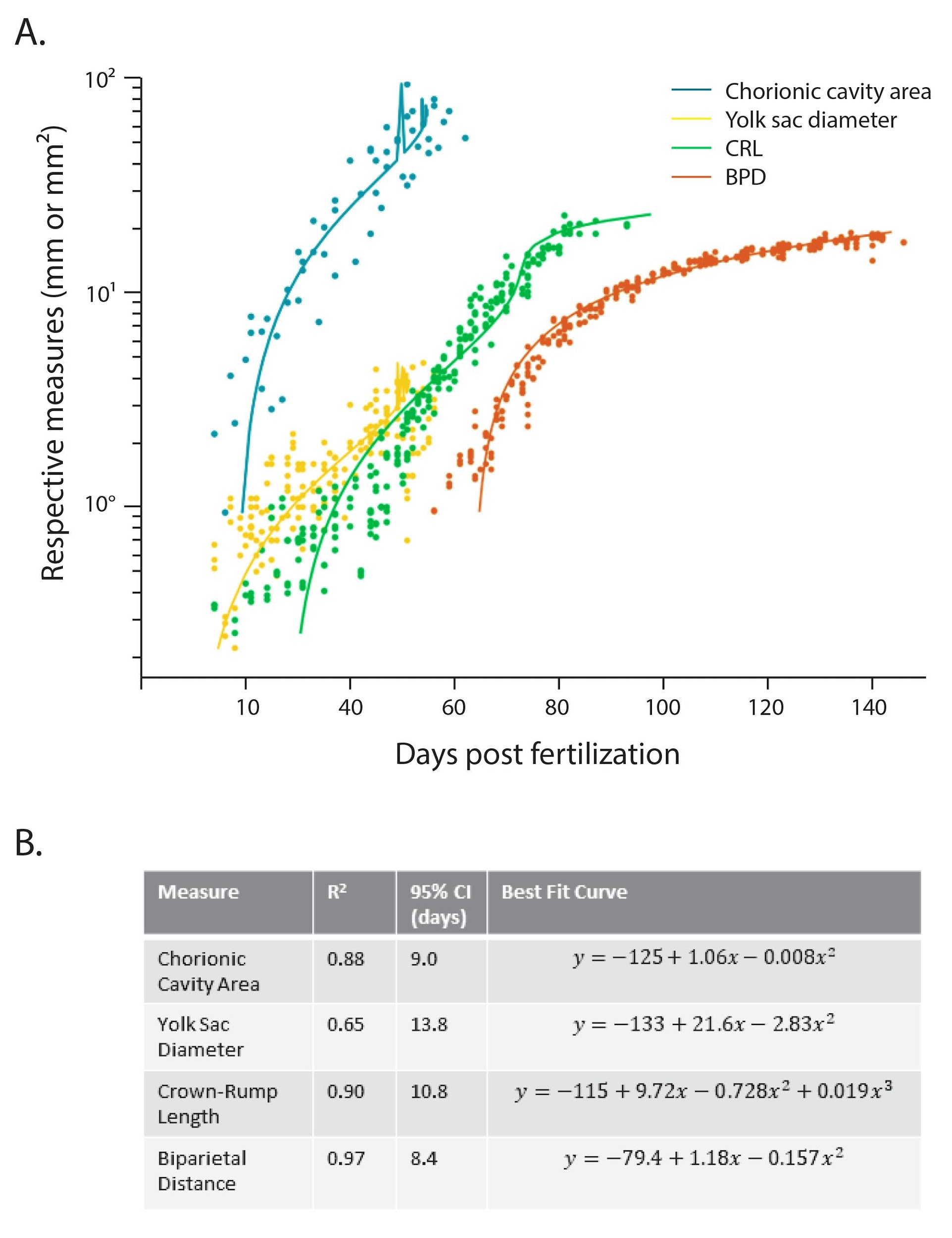
**
